# Pulmonary rehabilitation healthcare professionals understanding and experiences of the protected characteristics of service users: A qualitative analysis

**DOI:** 10.1177/14799731241307253

**Published:** 2025-01-14

**Authors:** Holly Drover, Sally J Singh, Mark W Orme, Enya Daynes

**Affiliations:** 1 Department of Respiratory Sciences, 4488 University of Leicester, Leicester, UK; 2 Centre for Exercise and Rehabilitation Science, National Institute for Health and Care Research (NIHR) Leicester Biomedical Research Centre, Respiratory, 4488 University Hospitals of Leicester NHS Trust, Leicester, UK

**Keywords:** Pulmonary rehabilitation, long COVID, breathlessness, chronic respiratory disease, heart failure, health inequalities

## Abstract

**Background:**

Health inequalities can affect access and uptake to pulmonary rehabilitation (PR). An individual’s protected characteristics (age, disability, gender reassignment, marriage and civil partnership, pregnancy and maternity, race, religion or belief, sex and sexual orientation) may contribute to health inequalities. Healthcare professionals (HCPs) experiences of the inclusivity and representativeness of PR services and knowledge of protected characteristics are unknown, however are vital for the identification and resolution of health inequalities. This qualitative study explored HCPs understanding of protected characteristics and their perception of the inclusivity, representativeness and equitable benefit of their PR services.

**Methods:**

Semi-structured qualitative interviews were conducted in person or via videoconferencing with HCPs involved in PR from two healthcare providers. Interviews were analysed using reflexive thematic analysis.

**Results:**

12 interviews were conducted with physiotherapists (*n* = 6), occupational therapists (*n* = 2), nurses (*n* = 2) and exercise physiologists (*n* = 2). Participants had a median (IRQ) age of 43 (13) and 75% (*n* = 9) were female. Four themes were generated. 1: ‘I don’t really know as much as I should’ [about protected characteristics]; 2: It’s uncomfortable collecting protected characteristics…; 3: ‘I don’t think [service users] are as representative as they could be’; 4: A conventional rehabilitation programme does not meet the needs of all.

**Conclusions:**

This study highlighted several challenges in HCPs understanding of protected characteristics and the representativeness of PR that must be addressed to ensure equity. Strategies, to understand barriers in accessing PR that limit representativeness should be explored.

## Introduction

Pulmonary rehabilitation (PR) is a highly evidence-based intervention consisting of exercise and education that is gold standard for individuals with chronic respiratory disease.^1–3^ UK guidance has recently suggested extending PR services to include other conditions such as long COVID and heart failure.^3^ It is recommended to offer PR to those that have a diagnosis of these conditions and experience breathlessness (Medical Research Council Dyspnoea score of ≥2).

These services in the UK, delivered by the National Health Service (NHS) are required to offer a comprehensive service for all individuals regardless of their protected characteristics.^4^ Protected characteristics are characteristics of an individual that are illegal to discriminate against as a result of the UK Equality Act 2010.^5^ The characteristics are age, disability, gender reassignment, marriage and civil partnership, pregnancy and maternity, race (which includes ethnicity), religion or belief, sex and sexual orientation.^5^ Protected characteristics are UK specific; however, many other countries have similar legislation.^5–13^

Knowledge of an individual’s protected characteristics (or equivalent) can facilitate assessment of health inequalities.^14,15^ Health inequalities are unfair, avoidable and systematic differences in individuals’ health status, access, experiences and outcomes of healthcare^16^ and are a major priority for healthcare providers globally.^17–19^ Health inequalities are driven by systemic factors,^20^ often resulting in individuals with certain characteristics experiencing greater inequality. A recent NHS England statement on health inequalities encouraged the collection of data, including some protected characteristics (age, ethnicity and sex), to ensure groups experiencing health inequalities can be identified and assist service development to increase inclusivity and representativeness.^15^ Recent scoping reviews found that protected characteristics and social determinants of health are not reported or are inconsistently reported in PR studies, resulting in health inequalities in this area remaining unclear.^21,22^ This is despite characteristics including age, deprivation, disability, gender, race/ethnicity and sex being shown to impact access, completion and outcomes of PR.^3,23–33^ The lack and inconsistency of reporting protected characteristics may be due healthcare professionals (HCPs) limited understanding of protected characteristics and their implications.

Currently, there is no research exploring PR HCPs knowledge of protected characteristics and their experiences of the inclusivity, representativeness and equitability of PR. Without this, services cannot accurately assess health inequalities and programmes cannot be effectively adapted to meet the needs of under-represented groups.

Therefore, the aim of this study was to explore HCPs understanding of protected characteristics and their perception of inclusivity, representativeness and equitable benefit of their PR services.

## Methods

This study has followed the COREQ reporting framework for qualitative research^34^ Ethical approval was granted by the University of Leicester Research Ethics Committee [reference:35782].

### Sampling

A purposive sampling approach was used with all qualified healthcare professionals (exercise physiologists, nurses, occupational therapists and physiotherapists) involved in PR at two centres (University Hospitals of Leicester NHS Trust (UHL) and Leicestershire Partnership Trust (LPT), Leicester, UK) eligible for the study. Participants from UHL were peripherally known to the interviewer as they were part of the same institution but different departments. There was no prior relationship with participants from LPT. Potential participants were approached by email in January 2023. Recruitment ceased once all eligible participants had been contacted and a representative sample was recruited.

### Data collection

Semi-structured interviews were conducted in person or via Microsoft Teams (based on participant’s preference) by one interviewer (HD), a female physiotherapist trained in qualitative interviewing. Interviews were chosen to allow an in-depth exploration of participants’ experiences of inclusivity, representativeness and equity in PR, which may be considered a sensitive subject. Only the participant and the interviewer were present during the interview. Participants were aware the study aimed to assess and improve health inequalities. The pilot tested topic guide covered knowledge of protected characteristics, the protected characteristics of service users attending PR services and the perceived equity of PR benefit (Supplement A). After discussing participants’ knowledge of protected characteristics, a list was provided for the remainder of the interview. Field notes were made during interviews. Interviews were audio-recorded and transcribed verbatim.

### Data analysis

The transcripts were analysed using Microsoft Word and Microsoft Excel following the stages of reflexive thematic analysis outlined by Braun and Clarke (familiarisation with the data, coding, generation of initial themes, development and review of themes, refining, defining and naming themes and writing up)^35^ using a phenomenological theoretical framework. Inductive coding was performed independently by two researchers (HD & MWO) to provide an alternative standpoint and themes and quotes discussed with the study team. Reflexive diaries were documented throughout the analysis process to reflect on how personal perspectives and social standpoints may shape the knowledge produced. These included reflecting on how the interviewers’ experience as a clinician allowed them to listen to participants with empathy and understanding.

## Results

12 out of the 16 (75%) eligible qualified healthcare professionals were recruited to the study (physiotherapists (*n* = 6), occupational therapists (*n* = 2), nurses (*n* = 2) and exercise physiologists (*n* = 2)) from January – March 2023 and gave informed consent. Interviews were conducted using Microsoft Teams (*n* = 8, 67%) or in person (*n* = 4, 33%) with a mean duration of 37 minutes (range 23-50 minutes). Participants had a median (IRQ) age of 43 (13) and 75% (*n* = 9) were female (see Table 1 for additional demographics). The demographics of the sample were representative of the department.

**Table 1.** A summary of the protected characteristics of participants.

Protected characteristic	
**Age, median (IRQ)**	43 (13), (1 participant preferred not to say)
**Disability n (%)**	Yes 0 (0%), No 11 (92%), prefer not to say 1 (8%)
**Ethnicity**	**Self-described ethnicity:** British Asian 1 (8%), Indian 1 (8%), White British 6 (50%), White British – English 1 (8%), White Czech 1 (8%), prefer not to say 1 (8%) **UK Census categories:** Asian/Asian British Indian 2 (17%), any other White background 1 (8%), White English/Welsh/Scottish/Northern Irish/British 7 (58%), White Irish 1 (8%), prefer not to say 1 (8%)
**Gender n (%)**	Woman 9 (75%), man 2 (17%), prefer not to say 1 (8%)
**Marriage and civil partnership status n (%)**	Married 10 (83%), divorced 1 (8%), prefer not to say 1 (8%)
**Pregnancy and maternity status n (%)**	Not currently pregnant and have not been pregnant in the last year 11 (92%), prefer not to say 1 (8%)
**Religion or belief n (%)**	None 7 (58%), Muslim 2 (17%), Christian 1 (8%) Hindu 1 (8%), prefer not to say 1 (8%)
**Sex n (%)**	Female 9, (75%), male 2 (17%), prefer not to say 1 (8%)
**Sexual orientation n (%)**	Heterosexual 11 (92%), prefer not to say 1 (8%)

UK Census Categories are as follows: White (English/Welsh/Scottish/Northern Irish/British, Gypsy or Irish Traveller, Irish, any other White background - option to self-describe), Mixed/Multiple ethnic groups (White and Black Caribbean, White and Black African, White and Asian, any other Mixed/Multiple ethnic background – option to self-describe), Asian/Asian British (Indian, Pakistani, Bangladeshi, Chinese, any other Asian background – option to self-describe), Black/African/Caribbean/Black British (African, Caribbean, any other Black/African/Caribbean background – option to self-describe), Other ethnic group (Arab, any other ethnic group – option to self-describe), prefer not to say.

**Figure 1.**
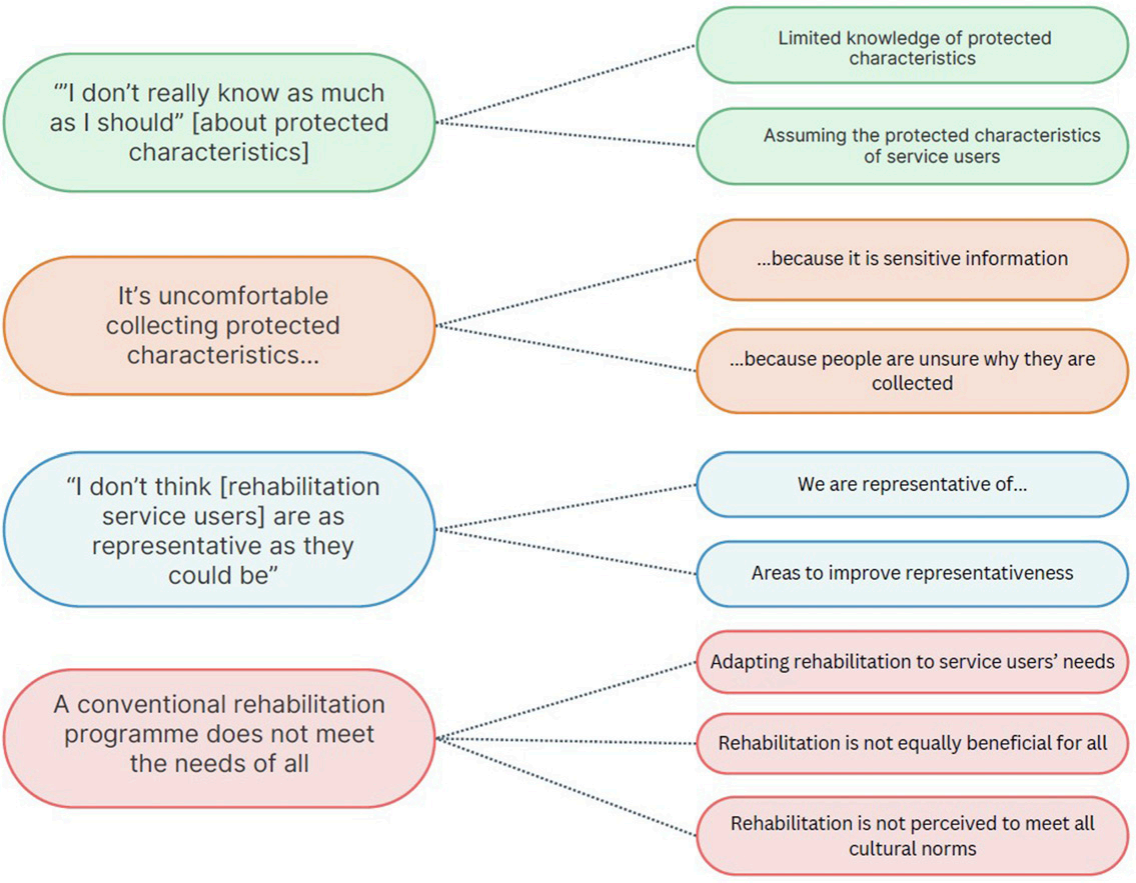
A visual representation of the themes and sub-themes.

Four themes were generated from the interviews: (1) *“I don’t really know as much as I should” about protected characteristics*; (2) *It’s uncomfortable collecting protected characteristics…;* (3) “*I don’t think [rehabilitation service users] are as representative as they could be*”; (4) *A conventional rehabilitation programme does not meet the needs of all* (Figure 1).

### Theme 1: “I don’t really know as much as I should” about protected characteristics

This theme explored HCPs perceived lack of knowledge about protected characteristics in their role and included the subthemes ‘*Limited knowledge of protected characteristics’* (1.1) and ‘*Assuming the protected characteristics of service users’* (1.2). Additional quotations are presented in Table 2.

**Table 2.** Additional quotations for Theme 1.

**Theme 1: “I don’t really know as much as I should” about protected characteristics**
**1.1 Limited knowledge of protected characteristics**
*it’s the shuttle walking test and obviously the management of the programme of it needing to be twice weekly for about 6 weeks *[participant 3, exercise physiologist]
*I’ve obviously heard the term, have an idea of what they are but I certainly don’t know in any detail any specific requirements or guidance if you like…I feel like I absolutely should *[participant 4, physiotherapist]
*I certainly feel like I need to understand that [gender reassignment] better...I probably don’t know a lot about it, to be honest with you *[participant 12, occupational therapist]
*would things like dyslexia…be considered a disability? ...I don’t know if that will come under it [*participant 12, occupational therapist]
*we see people with different disabilities…you know, mobility problems* [participant 11, occupational therapist]
*we do ask about sex…there is the option of them saying I identify as a man, woman or non-binary. So, they can be gender fluid *[participant 3, exercise physiologist]
**Subtheme 1.2: Assuming the protected characteristics of service users**
*because they’ve said I’m living with my husband…I don’t always even ask cause I’ve assumed I’m talking to a lady* [participant 11, occupational therapist]
*sometimes you see patients and they start telling you I saw this nurse, she was questioning all this [protected characteristics]. Then it was the doctor coming…he asked me the same questions, recorded it, then it was the senior doctor supervising who came and started all over again. Why do we have to collect the data that has been already collected* [participant 1, physiotherapist]
*It’s not that we actually ask if they’re married. We ask if they live with a spouse or a partner. Some of the patients will say, oh, yeah, I live with my partner, they could be married but they might just call them their partner. So, it’s not again that we ask, it’s more of an assumption [participant 3, exercise physiologist]*

#### Subtheme 1.1: Limited knowledge of protected characteristics

The majority of participants reported that they had limited, or inaccurate knowledge of the purpose of protected characteristics:

*that’s completely new for me *[participant 1, physiotherapist]

Limited knowledge was particularly evident around gender reassignment and marriage and civil partnership, with some participants surprised that marriage and civil partnership was a protected characteristic:

*marriage and civil partnerships are actually a bit of a surprise… I wouldn’t have thought that that would have been one *[participant 10, physiotherapist]

Several participants were unsure of the classification of disability as the protected characteristic:

*when you say disability…does that include physical and psychological? We ask about physical disabilities, we ask, do they feel that they have a disability or not? *[participant 10, physiotherapist]

Limited knowledge may have led to some participants voicing misunderstandings about protected characteristics including that they are a ‘new’ concept and a misunderstanding of sex and sexual orientation:

*we’re going to add in [collecting] these characteristics because we believe that times are changing *[participant 2, nurse]

*is there a difference between sex and sexual orientation? *[participant 9, physiotherapist]

Despite these misunderstandings, there was an evident appetite from HCPs to develop their understanding on this topic:

*I think that’s [protected characteristics] something that we need to learn more about as a team…I’ve asked if someone could do some training* [participant 12, occupational therapist]

#### Subtheme 1.2: Assuming the protected characteristics of service users

Some participants reported that they tended to assume service users’ protected characteristics. These are often collected as part of a telephone assessment which may have contributed towards this. Assumptions occurred for race and gender reassignment:

*where it [race] is a bit obvious, I go with what is on the [healthcare] system* [participant 1, physiotherapist]

*I tend to just assume that they are, their sex is from birth, their gender from birth* [participant 3, exercise physiologist]

Assumptions about service users’ protected characteristics may occur due to them expressing frustration that this information had been repeatedly collected, potentially perpetuating hesitancy of HCPs to ask:

“*they [service users] start telling you I saw this nurse, she was questioning all this [protected characteristics]. Then it was the doctor coming…he asked me the same questions…then it was the senior doctor supervising who came and started all over again*. *Why do we have to collect the data that has been already collected* [participant 1, physiotherapist]

Assumptions may also have occurred as HCPs felt uncomfortable asking the protected characteristics of service users, which is detailed in the next theme.

### Theme 2: It’s uncomfortable collecting protected characteristics…

This theme explored HCPs discomfort and perceived service users’ discomfort when collecting protected characteristics in PR *‘…because it is sensitive information’* (2.1) and *‘…because people are unsure why protected characteristics are collected’* (2.2). Additional quotations are presented in Table 3.

**Table 3.** Additional quotations for theme 2.

**Theme 2: It’s uncomfortable collecting protected characteristics…**
**Subtheme 2.1: …Because it is sensitive information**
*you feel like you’re delving in when you shouldn’t be…how would I feel if someone was just throwing these questions at me and I don’t think I particularly want that either?* [Participant 7, physiotherapist] *I had an argument with one of the patients…what does it have to do with her trying to exercise…So, I try to explain it and go the way, kind of like touching on the compliance. But it just did not work. And she put the phone down* [participant 1, physiotherapist]
**Subtheme 2.2:…** **B** **ecause people are unsure why they are collected**
*sometimes patients would be there like oh what do you need to know that for* [participant 3, exercise physiologist]
*you get a “What do you need to know that for?” I go, “Well, actually, that’s a good question,” but we sort of do* [participant 4, physiotherapist]
*what have some of these, or half of these to do with people doing exercise programmes* [participant 1, physiotherapist]
*[service users say] “why is she asking me that or just get on with it. I’ve come here for my lungs. Just talk about my lungs. What difference does it make if I’m this or this?* [Participant 10, physiotherapist]

#### Subtheme 2.1: …because it is sensitive information

This subtheme explored participants experiences of discomfort when asking service users their protected characteristics:

*I wouldn’t say I will feel comfortable doing it but I do it* [participant 9, physiotherapist]

This was particularly evident when asking perceived ‘intrusive’ characteristics for example, sexual orientation and gender reassignment:

*If I were attending something like a rehab programme and somebody said to me, you know, “Are you heterosexual?” ...I’d say, “Well, what business is it of yours?* [participant 4, physiotherapist]

*you don’t normally look at a stranger and suddenly ask them about their gender reassignment…that seems quite- like intrusive* [participant 7, physiotherapist]

Several participants reported that they perceived this discomfort resulted in service users terminating of their appointment:

*If it is a more sensitive subject, it closes the whole conversation and they might not want to then enrol in the programme* [participant 3, exercise physiologist]

Although this concern was raised by several participants, it is unknown whether all participants had experienced this, however it appeared to significantly impact the participants’ experiences and cautions around collecting protected characteristics. The perception of service users choosing to not uptake PR due to the collection of protected characteristics may impact how HCPs ask these questions to future service users.

#### Subtheme 2.2: …because people are unsure why they are collected

The subtheme of *‘…because people are unsure why they are collected’* describes HCPs not understanding the reasons for collecting protected characteristics and feeling uncomfortable justifying this to service users:

*it's just to collate information…that's how I kind of get out of it, it usually works…some people kind of give me a bit of a dodgy look…I don't want them to think of me differently* [participant 10, physiotherapist]

However, HCPs were more likely to collect protected characteristics if they knew why they were collected:

*if you’re aware that that information is being used to benefit the service then you're more likely to collect all that data* [participant 11, occupational therapist]

HCPs experienced service users querying the collection of characteristics, though an explanation often improved perceived service user comfort:

*as long that [why protected characteristics are collected] was relayed to the patient and they understood that, you know, this is why we collect this information, then most people I think would be fine* [participant 5, exercise physiologist]

Most participants, and some service users, did not perceive protected characteristics as clinically relevant, particularly marriage and civil partnership and sexual orientation:

*I can’t imagine that whether they’re married or in a civil partnership’s going to have any bearing on them- on us* [participant 8, physiotherapist]

*sexual orientation? ... it doesn't seem hugely important in terms of their rehab* [participant 12, occupational therapist]

*[service users say] “Why are you asking? What’s that got to do with my COPD?”* [participant 7, physiotherapist]

### Theme 3: “I don’t think [rehabilitation service users] are as representative as they could be”

This theme explored HCPs perceptions of the representativeness of PR services compared to the local population. There were protected characteristics where participants felt service users were representative (*‘We are representative of…’* (3.1)) and ‘*Areas to improve representativeness’* (3.2). Additional quotations are presented in Table 4.

**Table 4.** Additional quotations for theme 3.

**Theme 3: “I don’t think [rehabilitation service users] are as representative as they could be”**
**3.1 We are representative of…**
*they tend to be older people, retired people* [participant 12, occupational therapist]
*it tends to be a sort of- an older generation, you know, average age of around mid-60s* [participant 2, nurse]
*we’ve not had any pregnant or maternity patients* [participant 6, nurse]
*that goes with the age we tend to see kind of older people* [participant 12, occupational therapist]
*we rarely see- pre-, post-natal or pregnant women really [participant 7, physiotherapist]*
*so generally we would probably get kind of an older person…we don’t tend to see younger persons* [participant 6, nurse]
**3.2 ** **A** **reas to improve representativeness**
*it’s very rare to get people who aren’t White British [*participant 8, physiotherapist]
*I would say nearly 95% of the patients are White British* [participant 11, occupational therapist]
*I don’t think they’re [service users] as representative as what they could be* [participant 6, nurse]
*most of our patients are White British and that just doesn’t seem reflective of the population across Leicestershire* [participant 12, occupational therapist]
*Leicester is around 50% non-white...in all sort of rehab programmes…it isn’t generally a 50/50 split I would say in terms of white and non-white. So no, it probably isn’t fully representative of the diversity in Leicester* [participant 5, exercise physiologist]
*once a year we might get somebody who doesn’t entirely- doesn’t speak English. So it’s really quite rare* [participant 7, physiotherapist]
*we do have a lady in the class at the moment who has a Gujarati interpreter with her. But we’re still, yes, it’s still underrepresented, absolutely I can’t deny that* [participant 6, nurse]

#### Subtheme 3.1: We are representative of…

The subtheme titled *‘We are representative of…’* covered the participants perceptions that PR was representative of the target population in relation to age and pregnancy and maternity. Most PR deliverers reported that service users were usually older and without the protected characteristic of pregnancy and maternity, but this was representative of the population eligible for PR:

*because of the nature of the conditions, they’re usually diagnosed later in life* [participant 7, physiotherapist]

*zero pregnant or people on maternity leave* [participant 8, physiotherapist]

*because we have a much older population, you’re not going to see it [pregnancy and maternity] very much* [participant 3, exercise physiologist]

#### Subtheme 3.2: Areas to improve representativeness

This subtheme explored HCPs feelings in relation to under-representation of race and/or ethnicity and the spoken languages within PR services compared to the population they observe geographically.

Participants reported a predominantly White British population with few South Asian service users, which they did not feel was representative of the population of Leicester and Leicestershire:

*for the population of Leicester, which is almost like 50/50…against the South Asian…I would say that we’re still seeing mainly White males and females as opposed to South Asian* [participant 6, nurse]

*They tend to be…more White than you might imagine given where we’re delivering our services* [participant 4, physiotherapist]

HCPs displayed uncertainty around services users’ representativeness:

*I don't quite know why that's happening, but it doesn't feel like the patients we that we have coming to rehab or are kind of fairly representative of the local populations…it still doesn't feel equitable really across the population* [participant 12, occupational therapist]

HCPs reported that service users predominantly spoke English and that this was unrepresentative of the language diversity within the local population:

*we have had people before who, again not very often, but where English isn’t their first language…it doesn’t come up often* [participant 8, physiotherapist]

Many participants felt that language was a barrier to attending rehabilitation, particularly centre-based rehabilitation:

*if any of them don’t speak English or if they don’t have anyone that would translate for them, they tend to not turn up* [participant 3, exercise physiologist]

*they'd rather uptake take up the programme at home as opposed to classes because obviously in the classes you know they're not sure if there's someone else who can speak the language* [participant 10, physiotherapist]

Notably, participants compared a service users’ race, ethnicity and/or language to the local population, however compared a service users’ age or pregnancy and maternity status to the population eligible for PR. This suggests that participants were more confident about age and pregnancy and maternity demographics of those eligible for PR than race, ethnicity and/or language demographics. It is important to consider the demographics of those who are eligible for PR alongside the demographics of the local population, as local population statistics are unlikely to reflect that of the disease population.

The experiences of HCPs in this theme suggested that a conventional rehabilitation programme does not meet the needs of all service users (Theme 4).

### Theme 4: A conventional rehabilitation programme does not meet the needs of all

This theme explored HCPs experiences that a conventional rehabilitation programme consisting of supervised, centre-based aerobic exercise, resistance training and structured education, did not meet the needs of all service users. Participants expressed views that ‘*adapting rehabilitation to service users’ needs’* was necessary (subtheme 4.1), ‘*rehabilitation is not equally beneficial for all’* (4.2) and that ‘*rehabilitation is not perceived to meet all cultural norms’* (4.3). Additional quotations are presented in Table 5.

**Table 5.** Additional quotations for theme 4.

**Theme 4: A conventional rehabilitation programme does not meet the needs of all**
**4.1 Adapting rehabilitation to service users’ needs**
*if they have different [religious] beliefs, obviously we can’t really accommodate [them]…we don’t have the ability for that…there isn’t a female only or male only class* [participant 1, physiotherapist]
*age is not a discriminatory factor at all…you still can give them something to do the rehab* [participant 9, physiotherapist]
*we would hopefully address things in education point of view so, you know, in a fasting period for, you know, the different religious groups, we would talk about it* [participant 2, nurse]
*when you ask some of these [protected characteristic] questions…they’ve told us I need to do this, that or the other because of so and so and then we’d talk to them about how they could fit the rehabilitation program around it or adapt things* [participant 12, occupational therapist]
**4.2 ** **R** **ehabilitation is not equally beneficial for all**
*the higher the education more likely you would engage. Then, it’s the social aspect, connected with your work, money and what is available…poorer families, unemployed, on benefits are less likely to fully complete* [participant 1, physiotherapist]
*so if they’ve got greater opportunities, they’re more likely to see a better benefit, aren’t they?* [Participant 5, exercise physiologist]
*some of our patients are coming to or being referred to us after so many years since they had the condition…It’s not that we can’t do things with them later, we can. But the potential benefit might be slightly minimised because of the impact of the condition* [participant 1, physiotherapist]
**4.3 ** **R** **ehabilitation is not perceived to meet all cultural norms**
*in sort of local communities, it’s the importance of accepting sort of different types of healthcare or medication or treatment…some subjects are just taboo* [participant 2, nurse]
*sometimes explaining to people with a different language about breathlessness, I don’t know that they have got the understanding* [participant 11, occupational therapist]
*I had a South Asian lady who really didn’t understand what I was talking about when I was talking about breathlessness. She had no idea. It wasn’t really a word she’d used* [participant 6, nurse]
*it’s quite common for the family to take on a caring role for their elders. So whether kind of promoting them to kind of exercise and do more for themselves when they’re perhaps, a clash of well you’re older now we look after you, it’s our time to look after you* [participant 12, occupational therapist]
*the perception of what that [exercise] is or how that sits within their sort of life experience or, you know, where it sits culturally, …you sort of wonder about…whether exercise is a more acceptable thing for some people than others* [participant 4, physiotherapist]

#### Subtheme 4.1: Adapting rehabilitation to service users’ needs

Participants did not feel that rehabilitation was fully accessible to all as a conventional programme:

*with it being exercise-based rehab, there’s a level of ability that they have…you wouldn’t necessarily see many people that would fall under the sort of disability category* [participant 5, exercise physiologist]

However, participants did feel that PR could be adapted, particularly for disability:

*we make reasonable adjustments for everyone who’s accessing [the service]* [participant 6, nurse]

*people come in obviously, you know, walking aids, different mobility issues. Sometimes you end up doing total adaptation to seated programme* [participant 9, physiotherapist]

Some participants felt that collecting protected characteristics provided an opportunity to measure if PR is accessible for all and make adaptations for under-represented groups based on this:

*you can monitor that people are not being excluded to get an idea of who's accessing the services. So we can see…you're not excluding certain groups completely. And plan services as well around what's needed* [participant 11, occupational therapist]

#### Subtheme 4.2: Rehabilitation is not equally beneficial for all

Participants did not believe that PR was equally beneficial for all service users. For example, HCPs did not perceive all service users to have equal opportunities to exercise outside of sessions which resulted in unequal improvements:

*it might come down to opportunities to exercise outside of coming to the sessions as well...if you are someone that comes from a certain background [lower socioeconomic status], you might have more chance to stay active…whereas other people that session that they attend with us might be the only chance that they get* [participant 5, exercise physiologist]

Although it is not a protected characteristic, participants also expressed views that socioeconomic status impacts PR outcomes:

*it makes a difference when people are educated. They can take on board the education… often, they'll go out and buy some exercise equipment so they can carry on exercising at home* [participant 11, occupational therapist]

#### Subtheme 4.3: Rehabilitation is not perceived to meet all cultural norms

This final subtheme explored HCPs perceptions that PR may not meet cultural norms. Several participants discussed their perceptions that accessing management may be more difficult in certain cultures:

*for the female part in the Asian population where it would be typical at that age…it’s the woman running the house…we may not get as many of those patients because they will still irrespective run the house* [participant 1, physiotherapist]

Participants also discussed that cultural perceptions and understanding of breathlessness may vary by language and ethnicity:

*it’s sometimes maybe lost in translation as to the severity of their breathlessness…some patients, because of their condition, will push themselves only so far and think this is it. I must stop because it’s brought on a sensation of breathlessness* [participant 3, exercise physiologist]

The perceptions of the acceptability of exercise in different cultures was discussed by HCPs. Many participants believed that exercise was less acceptable in certain cultures than others:

*I come from an Asian background, so culturally if I asked my mum to exercise, it wouldn’t happen…culture has a huge influence because they feel it’s wrong to do something deviated from the normal* [participant 9, physiotherapist]

This meant that the conventional exercise-based programmes were not perceived to meet the needs of all cultures.

## Discussion

This qualitative study has explored HCPs understanding of protected characteristics and their perceptions of the inclusivity, representativeness and equitable benefits of PR within Leicestershire. Participants had limited knowledge resulting in discomfort when assessing protected characteristics, however intended to increase their knowledge. Limited knowledge may lead to misunderstandings and assumptions of service users protected characteristics. Training for HCPs could improve the understanding and assessment of protected characteristics in relation to health inequalities.^36,37^ Training sessions could include importance and approaches of data collection, how to analyse the data to support service improvement and communicating to service users.^38^

Unease experienced by HCPs and perceived discomfort experienced by service users when collecting protected characteristics was frequently discussed by participants. A recent scoping review identifying approaches in healthcare for monitoring health inequalities reported that staff were reluctant to collect health inequality data (including protected characteristics).^39^ Comparatively, this was a result of staff reluctance due to a lack of knowledge and concerns of causing offense.^39^ Participants viewed sexual orientation and gender reassignment as particularly sensitive information, but research has shown that the majority of service users are comfortable answering these questions within healthcare.^40,41^ All characteristics add value in assessing health inequalities, and the ways to address them.^42^

Most participants perceived that the majority of service users identified as White British which they felt was unrepresentative of the local population. In Leicester (UK), 33.2% of the population identifies as White English/White Welsh/White Scottish/White Northern Irish/White British compared to the national average of 73.5%. 43.4% identify as Asian/Asian British/Asian Welsh compared to the national average of 9.7%.^43^ The perception that the majority of service users attending PR were White British is consistent with the UK National Respiratory Audit Programme’s (NRAP) report that 82.5% of service users attending PR are White British^32^; though this report includes data on people with Chronic Obstructive Pulmonary Disease (COPD), which is predominantly diagnosed in White British individuals^44^ and underdiagnosed in ethnic minority groups.^45,46^ There is a reported underdiagnosis of respiratory diseases among non-White individuals due to race adjustment equations in pulmonary function tests^47,48^ and access to healthcare.^45,46^ In one study in London (UK), the prevalence of diagnosed COPD was 1.55% in White individuals, 0.78% in Asian individuals and 0.58% in Black individuals^44^ which may present a barrier to referral to PR. However, within UHL access to PR is not dependent on a diagnosis which should improve access to this key intervention. Similarly, in New Zealand and the United States, Maori and Black individuals are significantly less likely to attend PR than European and non-Hispanic White individuals respectively.^25,33,49^ The differences in attendance could not be accounted for using regional variation in service provision.^25^ Several studies suggest ethnic concordance between service users and HCPs may increase a service users’ trust and comfort,^50–52^ potentially making a service user more likely to attend PR. This suggests strategies such as adapting programmes for under-represented service users and striving for a diverse workforce may be needed to improve the representativeness of PR.

Several participants discussed their perceptions that PR is not inclusive for all, particularly in relation to the cultural acceptability of exercise. An individual’s culture includes their values, beliefs and conventions that influence their behaviour and are shared by a group of people.^53^ Exercise is key component of rehabilitation; however, it has been reported that in South Asian communities, exercise can be perceived as detrimental to an individual’s reputation,^54–56^ and impacting family honour, whereby beliefs prioritise family duties over independence and exercise.^54–56^ This suggests adaptations to optimise cultural accessibility are required to ensure inclusivity for all, whilst maintaining effectiveness.

This study recruited participants from two NHS trusts in Leicestershire (UK), therefore, the experiences of the HCPs in this study are not transferrable to HCPs at other NHS trusts, or in different countries. However, whilst HCPs experiences will differ depending on location, health inequalities are a global challenge and therefore this work could offer insight into potential challenges. Although this study focused on protected characteristics, there are additional health inequalities that should also be considered, such as socioeconomic status, and some protected characteristics that may be less influential in the context of PR. Frameworks such as INCLUDE^57^ and the allied health professionals health inequality action framework^58^ may support the exploration of the most relevant health inequalities. The sample consisted of 75% of the eligible population and included a range of professions working in PR, widening the scope of experiences explored. Participants consisted of predominantly White, female participants without a disability and although this reflects the demographics of the professions interviewed,^59,60^ it may have resulted in similar positionality, views and experiences. To increase the rigour of the study, codes and themes were discussed with the study team to reflect on data that may have been overrepresented or overlooked. Though these findings have limited transferability, they highlight the importance of protected characteristics, and supporting HCPs to explore these in a way that is safe and acceptable in order to evaluate health inequalities.

In conclusion, this qualitative study has highlighted several challenges in HCPs knowledge and understanding of protected characteristics and the inclusivity and representativeness of PR that must be addressed to ensure equity for individuals eligible for PR. Participants reported a limited knowledge of protected characteristics, therefore education sessions for HCPs to improve their understanding, communication to service users and data analysis to support service improvement could be beneficial. Conventional PR programmes were not viewed to be representative of the local population or inclusive of all eligible service users by HCPs. Strategies to understand barriers in accessing PR that limit representativeness should be explored to improve the inclusivity and representativeness of PR.

## Supplemental Material

Supplemental Material - Pulmonary rehabilitation healthcare professionals understanding and experiences of the protected characteristics of service users: A qualitative analysis Supplemental Material for Pulmonary rehabilitation healthcare professionals understanding and experiences of the protected characteristics of service users: A qualitative analysis in China by Holly Drover, Sally J Singh, Mark W Orme and Enya Daynes in Respiratory Disease.
