# Supplementary material for: Pulmonary rehabilitation healthcare professionals understanding and experiences of the protected characteristics of service users: A qualitative analysis
Source: Chron Respir Dis. 2025 Jan 14;22:14799731241307253. doi: 10.1177/14799731241307253 (PMC11733882; doi:10.1177/14799731241307253)
Supplement: Supplemental Material - Pulmonary rehabilitation healthcare professionals understanding and experiences of the protected characteristics of service users: A qualitative analysis [file sj-pdf-1-crd-10.1177_14799731241307253.pdf]

Supplementary Material

Supplement A: Topic Guide

INTERVIEW TOPIC GUIDE

Welcome and introduction

Confirm consent to continue and to audio-record the interview.

Let them know that no personal identifiable data will be recorded and a participant number will be allocated to them

- **Could you tell me about your role within pulmonary rehabilitation?**
  - Professional background, experience, specific duties
- **What do you know about the UK's protected characteristics?**
  - Can you name as many of the protected characteristics as you can? What does each of them mean to you? How would you define or explain them to someone?
  - ***Provide handout with list for remainder of interview***: Do any of the protected characteristics listed surprise you? Is there anything you thought might be on there that isn't? Anything you think might have been overlooked? [Why/why not]
- **Can you describe the people you tend to see attending pulmonary rehabilitation?**
  - In relation to the protected characteristics
  - Why do you think there is/isn't representation in [each protected characteristic]?
  - How representative do you think pulmonary rehabilitation attendees are to the wider population? Wider patient population? Wider Leicestershire population? [Why/why not]
- **What have been your experiences of seeing (or not seeing) under-represented patients in pulmonary rehabilitation?**
  - Referral, attendance, uptake, retention, effectiveness, enjoyment
  - Does this need to be improved? How could this be improved? [Why/why not]
  - What about your experiences of [protected characteristics not mentioned]?
- **What is your experience of breathlessness management in under-represented groups?**
  - Any adaptations?
  - Does it present differently?
- **Which protected characteristics do you collect information about for pulmonary rehabilitation?**
  - Why these? Why not others?
  - How do you collect them? What options are provided?
  - How do people respond to these questions? [Positive/negative]
- **What do you think about collected information on all protected characteristics as part of pulmonary rehabilitation?**
  - What do you think the information could be used for?
  - Is there anything that would make you more/less likely to collect the information?
  - What might some of the challenges be? How can they be overcome?
  - Do you think collecting this information should be part of research/usual care/audit? [Why/why not]

Anything not covered? Is there anything that we haven't covered in the interview that you think we should know or think about?

Check that they're ok and there are no safety concerns.

Closing and thanks - check that the participant is still happy for you to use all the information provided and offer the possibility to erase sections of the recording.

Thank them for their time and contribution.
